# The Rolling Stone: Bouveret Syndrome Requiring Open Gastrotomy After Failing Electrohydraulic Lithotripsy

**DOI:** 10.7759/cureus.39470

**Published:** 2023-05-25

**Authors:** Zarian Prenatt, Subin Chirayath, Janak Bahirwani, Rodrigo Duarte-Chavez

**Affiliations:** 1 Internal Medicine, St. Luke's University Health Network, Bethlehem, USA; 2 Gastroenterology, St. Luke's University Health Network, Bethlehem, USA

**Keywords:** gallstones, electrohydraulic lithotripsy, gastric outlet obstruction, gallstone diseases, gastric outlet obstruction (goo), endoscopic electrohydraulic lithotripsy, biliary-enteric fistula, gallstone ileus, bouveret syndrome

## Abstract

Bouveret syndrome (BS) is an extremely rare form of gallstone ileus where a stone travels through a biliary-enteric fistula and causes gastric outlet obstruction. A 92-year-old male presented with gastric outlet obstruction secondary to an impacted gallstone in the duodenal bulb seen on imaging. Endoscopic therapy failed twice due to the immense gallstone size, and an open gastrotomy was required to remove the stone. The procedure was successful; however, the patient, unfortunately, passed away days after the operation due to other hospital illnesses. BS should be considered in patients with advanced age and significant comorbidities presenting with gastric outlet obstruction.

## Introduction

Bouveret syndrome (BS) is characterized by gastric outlet obstruction secondary to an impacted gallstone in the duodenal bulb [1]. This is a unique form of gallstone ileus where a gallstone enters the enteric system through an acquired biliary-enteric fistula [2]. Spontaneous biliary-enteric fistulas are rare, but over 90% occur as a complication of cholelithiasis or choledocholithiasis [3]. Fistula formation and gallstone passage in BS are due to adhesions between the gallbladder and bowel wall from inflammation causing increased intraluminal pressure with subsequent wall ischemia and perforation [4]. Gallstone ileus is an extremely uncommon complication, seen in only 0.3-0.5% of patients with cholelithiasis, and BS is the rarest form of gallstone ileus, as it represents 1-3% of these cases [5]. There are approximately 300 cases of BS reported in the literature since first being described in 1986 by French physician Leon Bouveret [4,6]. We present a case of BS that failed multiple attempts of endoscopic electrohydraulic lithotripsy (EHL) due to the large size of the stone and ultimately required open gastrotomy.

## Case presentation

**Figure 1:**
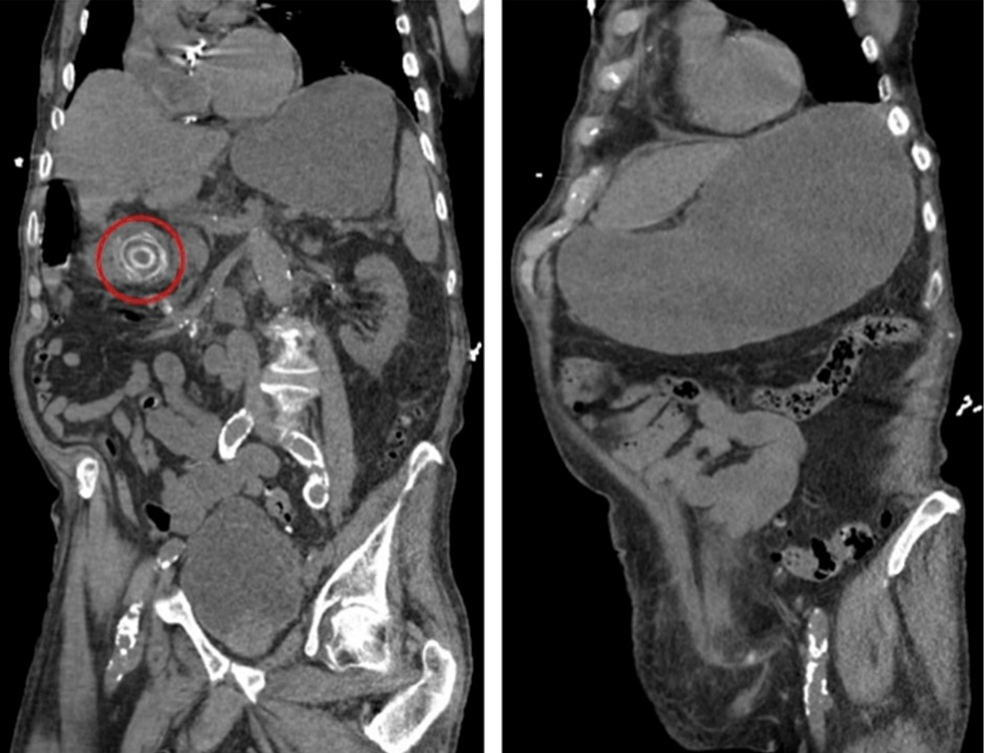
Initial computed tomography of the abdomen revealed (A) a large gallstone in the first portion of the duodenum causing (B) gastric outlet obstruction.

**Figure 2:**
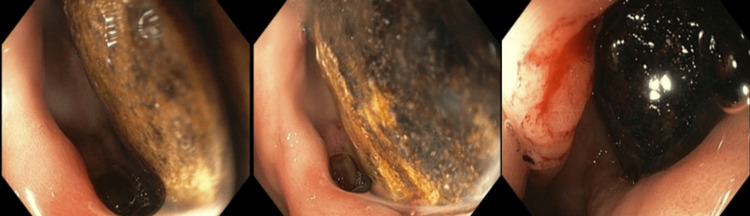
Upper endoscopy showed a large obstructing gallstone in the duodenal bulb occupying approximately 95% of the luminal area.

A 92-year-old male with a past medical history only notable for dementia presented to the emergency department with acute onset abdominal pain, nausea, and vomiting. Vital signs on arrival revealed a temperature of 97.4°F, heart rate of 110 beats per minute, respiration rate of 18 breaths per minute, blood pressure of 100/88 mmHg, and pulse oximetry of 97% on room air. Physical examination revealed a soft, non-tender, and non-distended abdomen with decreased bowel sounds. Initial labs were notable for an elevated white blood cell count at 17.5 thousand/uL, elevated lactic acid at 2.4 mmol/L, and elevated creatinine at 2.35 mg/dL without a known baseline. His liver enzymes were within normal limits. Urine and blood cultures were positive for Enterococcus faecalis, confirming the source of his sepsis. A computed tomography (CT) scan of the abdomen revealed a large gallstone causing gastric outlet obstruction (Figure 1). Gastroenterology was consulted, and upper endoscopy revealed a large obstructing gallstone in the duodenal bulb (Figure 2). EHL was performed to fragment the stone; however, this was unsuccessful, as it only fragmented a small portion of the gallstone after 4,000 pulses. A large remnant of the gallstone remained in the duodenal bulb, and the patient underwent a repeat endoscopy two days later with another unsuccessful attempt at fragmentation using EHL due to the large size of the gallstone.

**Figure 3:**
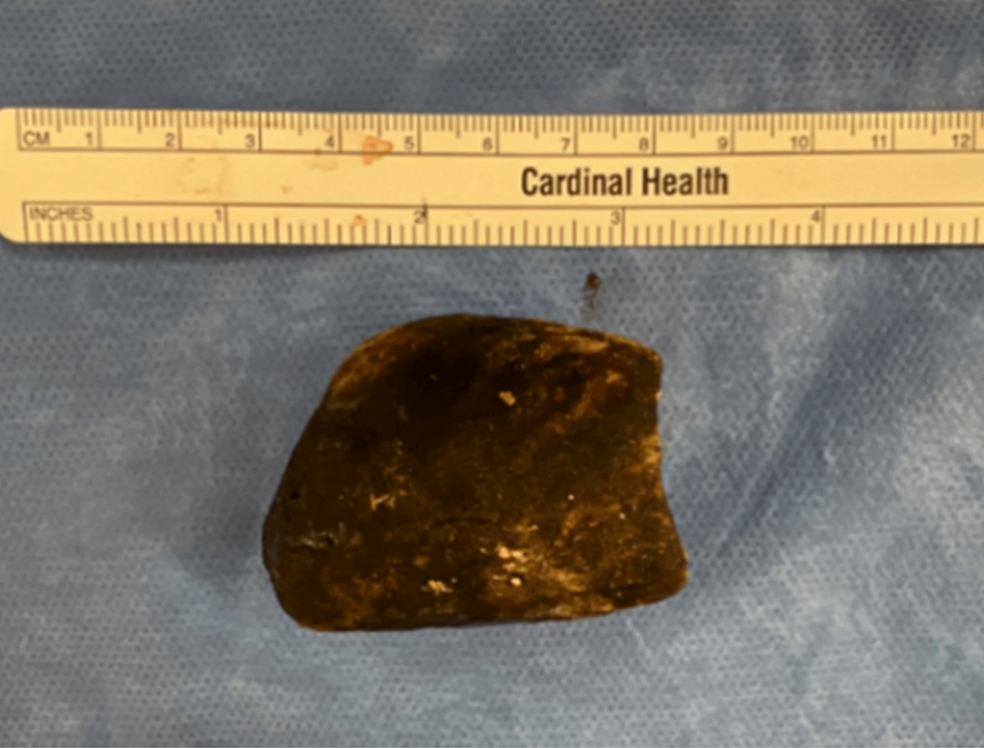
A 5 cm x 4 cm x 2.5 cm gallstone extracted via surgical gastrotomy after causing gastric outlet obstruction.

General surgery was consulted for consideration of surgical extraction, and the patient underwent exploratory laparotomy. An extremely dense and immobile mass was found lodged within the proximal second portion of the duodenum, which was unable to be milked proximally into the gastric lumen. The lesser sac was entered to improve exposure. The dilated duodenum was exposed, and the obstructing gallstone was milked proximally into the gastric lumen. A longitudinal gastrotomy was made in the gastric body revealing a 5 cm x 4 cm x 2.5 cm gallstone (Figure 3) and a cholecystoduodenal fistula. The stone was successfully extracted. Successful intraluminal passage of a 14 French enteric tube through the gastrotomy confirmed that the patient's obstruction was resolved. A gastrojejunal feeding tube was placed to provide enteral nutrition distal to the cholecystoduodenal fistula and gastrotomy. Postoperatively, the patient remained intermittently delirious and hypotensive with labile urine output, which responded to conservative fluid resuscitation. Unfortunately, he was noted to be apneic and passed away four days following his operation.

## Discussion

Gallstones are an extremely common cause of digestive disorders in the United States. It has been estimated that more than 6 million males and 14 million females in the United States aged 20-74 have gallstones [7]. Gallstones are more prevalent with increasing age, obesity, and chronic illnesses. These stones are usually asymptomatic, but sometimes they can lead to complications, including cholecystitis, choledocholithiasis, ascending cholangitis, and acute pancreatitis. More rare complications include the development of a biliary-enteric fistula and gallstone ileus [8].

Gallstone ileus is characterized by small bowel obstruction due to the impaction of a gallstone that has traveled through a biliary-enteric fistula. Although gallstone ileus is uncommon, accounting for 1% of cases of small-bowel obstruction in the general population, the incidence increases to 25% in patients older than 65 years presenting with a small-bowel obstruction without the presence of a hernia [9,10]. It affects females up to six times more often than males [9]. The terminal ileum is the most commonly obstructed location (50-70%) since it is the narrowest [9]. Other sites of obstruction are less common and include the distal jejunum (9%), colon (4%), rectum (4%), and duodenum (1-3%) [9].

In BS, a rare variant of gallstone ileus, proximal movement of a gallstone into the duodenum occurs leading to gastric outlet obstruction [11]. Approximately 1-3% of duodenal obstructions are due to BS [9]. Similar to gallstone ileus, there is a higher incidence of BS in women compared to men, with a female-to-male sex ratio of 1.86 [11]. This finding is due to the higher incidence of gallstones in women, which has been attributed to the cholestatic effects of estrogen [12]. BS also predominates in the elderly population with a mean age of 74 years [13]. BS typically presents with nonspecific symptoms, such as nausea, vomiting, and abdominal pain, as demonstrated in our patient [11]. The diagnosis of BS is based on clinical findings in combination with a duodenal obstruction and visualization of a stone by imaging or endoscopy [14]. Advanced age with comorbidities, delayed diagnosis due to non-specific presentations, and complex management contribute to the high mortality rate seen in BS (12-30%) [4]. Although his gender makes this an atypical presentation, our patient likely developed BS as a result of his advanced age in the setting of his numerous hospital illnesses.

Treatment options for BS can be broadly divided into non-surgical and surgical approaches. Endoscopic mechanical lithotripsy, endoscopic laser lithotripsy, extracorporeal shock wave lithotripsy, and intracorporeal EHL are all non-surgical options used to fragment the obstructing gallstone into smaller pieces [5]. Retrieval of gallstones using endoscopy with or without lithotripsy is preferred in high-risk elderly patients with significant comorbidities and should always be attempted as the first-line treatment [15]. Endoscopic treatment can be technically challenging and time-consuming with success rates in case series reported to be less than 10% [15]. Some challenges with lithotripsy include the need for prolonged sessions, repeat procedures, and the risk of perforation or converting proximal gallstones into distal stones after fragmentation [11]. The success rate of endoscopic removal is dependent on gallstone size with larger stones being more difficult to remove [11]. Case reports have demonstrated that stones larger than 2.5 cm are more challenging to extract endoscopically [11]. Additionally, these gallstones often have a dense outer shell with a soft core making the fragmentation process more difficult [11]. We first attempted endoscopic EHL in our patient. This approach, unfortunately, failed two times, which we attribute to the immense size and hard outer shell of the gallstone.

Given the low success rate of endoscopic retrieval, the mainstay of treatment for BS is surgery. Surgery is typically performed using two different approaches, and the choice depends on the patient’s age, comorbidities, and clinical status [16]. One surgical approach is enterolithotomy or gastrotomy combined with fistula repair and cholecystectomy as a definitive one- or two-step procedure [16]. An advantage to this option is the prevention of complications related to a non-definitive biliary procedure, such as cholangitis, cholecystitis, recurrent ileus, gastrointestinal bleeding, and gallbladder carcinoma [17]. Unfortunately, the majority of patients with BS are elderly with significant comorbidities, which increases the risk of postoperative complications and mortality when utilizing this definitive approach [5]. In these higher-risk patients, another surgical option exists where enterolithotomy or gastrotomy with stone extraction is performed alone with fistula repair and cholecystectomy reserved for patients who experience future symptoms [16]. Advocates for this approach argue the need for a definitive biliary procedure due to the high likelihood of spontaneous closure of the fistula in the presence of a patent cystic duct. The literature reports that only 10% of patients eventually require additional surgery for biliary symptoms, and there is a lack of evidence to support the theoretical risk of developing gallbladder carcinoma with a persistent fistula [15]. Additionally, the mortality rate is lower at 12% with simple stone extraction compared to the 20-30% mortality rate associated with a definitive approach [18]. Although we pursued the safer surgical approach for our patient, he, unfortunately, passed away days after the procedure during the recovery period. We believe this is not a direct consequence of his operation, but rather multifactorial in the setting of his advanced age with significant comorbidities, including urosepsis and renal dysfunction.

## Conclusions

This case underscores the complexities involved in managing BS. Endoscopic management is the preferred initial treatment; however, this has a low success rate, as evidenced in our case. Surgery is the mainstay of treatment and carries a higher mortality risk, particularly in elderly patients with significant comorbidities. Further studies are needed to explore optimal management strategies and improve outcomes in patients with BS.

## References

[1] Pickhardt PJ, Friedland JA, Hruza DS, Fisher AJ (2003). Case report. CT, MR cholangiopancreatography, and endoscopy findings in Bouveret's syndrome. AJR Am J Roentgenol.

[2] Caldwell KM, Lee SJ, Leggett PL, Bajwa KS, Mehta SS, Shah SK (2018). Bouveret syndrome: current management strategies. Clin Exp Gastroenterol.

[3] Pickhardt PJ, Bhalla S, Balfe DM (2002). Acquired gastrointestinal fistulas: classification, etiologies, and imaging evaluation. Radiology.

[4] Haddad FG, Mansour W, Deeb L (2018). Bouveret's syndrome: literature review. Cureus.

[5] Wang F, Du ZQ, Chen YL, Chen TM, Wang Y, Zhou XR (2019). Bouveret syndrome: a case report. World J Clin Cases.

[6] Lowe AS, Stephenson S, Kay CL, May J (2005). Duodenal obstruction by gallstones (Bouveret's syndrome): a review of the literature. Endoscopy.

[7] Everhart JE, Khare M, Hill M, Maurer KR (1999). Prevalence and ethnic differences in gallbladder disease in the United States. Gastroenterology.

[8] Abou-Saif A, Al-Kawas FH (2002). Complications of gallstone disease: Mirizzi syndrome, cholecystocholedochal fistula, and gallstone ileus. Am J Gastroenterol.

[9] Reisner RM, Cohen JR (1994). Gallstone ileus: a review of 1001 reported cases. Am Surg.

[10] Gan S, Roy-Choudhury S, Agrawal S, Kumar H, Pallan A, Super P, Richardson M (2008). More than meets the eye: subtle but important CT findings in Bouveret's syndrome. AJR Am J Roentgenol.

[11] Mishra A, Jain A, Lal P, Hadke NS (2013). Bouveret syndrome: a case report and review. J Gastroint Dig Syst.

[12] Ferhatoğlu MF, Kartal A (2020). Bouveret's syndrome: a case-based review, clinical presentation, diagnostics and treatment approaches. Sisli Etfal Hastan Tip Bul.

[13] Bateson MC (1984). Gallbladder disease and cholecystectomy rate are independently variable. Lancet.

[14] Moschos J, Pilpilidis I, Antonopoulos Z (2005). Complicated endoscopic management of Bouveret's syndrome. A case report and review. Rom J Gastroenterol.

[15] Nickel F, Müller-Eschner MM, Chu J, von Tengg-Kobligk H, Müller-Stich BP (2013). Bouveret's syndrome: presentation of two cases with review of the literature and development of a surgical treatment strategy. BMC Surg.

[16] Qasaimeh GR, Bakkar S, Jadallah K (2014). Bouveret's syndrome: an overlooked diagnosis. A case report and review of literature. Int Surg.

[17] Liew V, Layani L, Speakman D (2002). Bouveret's syndrome in Melbourne. ANZ J Surg.

[18] Sica GS, Sileri P, Gaspari AL (2005). Laparoscopic treatment of Bouveret's syndrome presenting as acute pancreatitis. JSLS.

